# Enhancing Health Care in the Veteran Community Through Synergistic Research Funding

**DOI:** 10.3389/fpsyt.2021.541889

**Published:** 2021-02-18

**Authors:** Michelle D. Lane, Kristy B. Lidie, Ray F. Santullo, Stephen J. Dalal

**Affiliations:** Congressionally Directed Medical Research Programs (CDMRP), U.S. Army Medical Research and Development Command (USAMRDC), Fort Detrick, MD, United States

**Keywords:** research and development, veteran health, veterans, mental health, biopsychosocial research

## Abstract

The veteran population faces myriad health burdens, particularly regarding mental health. As veterans age, combined genetic, environmental, and biochemical factors with natural biological processes may increase their susceptibility to mental health disorders as well as neuropsychiatric and dementia-related disorders that present as persistent cognitive impairment. Multi-organizational, multidisciplinary research partnerships help explore relevant evidence-based methodologies and create a two-way continuum between basic science and clinical application to address veterans', often complex, health care needs. The Congressionally Directed Medical Research Programs (CDMRP), a global funding organization located within the U.S. Army Medical Research and Development Command (USAMRDC), fosters novel approaches to biomedical research in response to the expressed needs of stakeholders and, as directed by Congress, many CDMRP programs focus on topics that are relevant to the health care of veterans. The CDMRP's foundation as a research management organization includes a two-tier review process and fully integrates consumer advocates. The CDMRP complements the U.S. Department of Veterans Affairs (VA) research through collaborative partnerships and synergistic award mechanisms tailored to areas of greatest need. Continued partnerships between the VA and CDMRP can facilitate translation of basic research to clinical application and enhance health care in the veteran community. This perspective highlights the need for research to address mental health issues affecting the veteran community, describes how the CDMRP integrates veterans into its processes, and discusses how the CDMRP's processes and collaborations with the VA have the capacity to improve mental health care for veterans.

## Introduction

Service members and veterans experience military-specific stressors that require focused research. Results from research conducted with the general population, therefore, may not translate to these subpopulations. Research and collaboration to address mental health conditions and to promote resilience among service members and veterans are priorities within the Department of Defense (DOD) and VA (1).

The veteran population faces numerous and significant health burdens. Specifically, veterans experience traumatic brain injury (TBI) and mental health disorders at disproportionate rates compared to their non-veteran counterparts (2). In a large Veterans Health Administration primary care sample, the prevalence of post-traumatic stress disorder (PTSD), substance use disorder, anxiety, and serious mental illness was higher than that of the general population (3). Individuals with PTSD often experience other physical and mental health comorbidities that increase the complexity of treatment and the burden on patients. Among Operation Enduring Freedom/Operation Iraqi Freedom veterans, alcohol dependence and PTSD are particularly comorbid (4). As veterans age, environmental conditions, genetics, and biochemistry, combined with naturally occurring biological processes, may make them more susceptible to neuropsychiatric and dementia-related disorders as well (5). For example, evidence suggests that veterans with PTSD are at increased risk of developing dementia, though the mechanisms linking these disorders are not fully understood (6). Mental health stigma, which may be unintentionally amplified by military culture, is a barrier to care that further complicates assessment and treatment of mental health disorders (7). The complex interplay among these factors highlights the need for focused research to address the health care needs of veterans.

Multi-organizational, multidisciplinary research partnerships fostered by the CDMRP are well-positioned to address the many complexities with veteran healthcare. The purpose of this perspective is threefold: (1) to describe how key facets of the CDMRP's program cycle integrate veterans and veteran health care perspectives, (2) to discuss the importance of collaborative partnerships and synergy between the CDMRP and the VA when reviewing and conducting veteran-focused scientific research, and (3) to promote the bi-directional translation of basic science to clinical application to improve veteran mental health.

## CDMRP Overview

The CDMRP is a directorate located within the USAMRDC that manages over 35 biomedical research programs, as directed by Congress (see Table 1). The mission of the CDMRP is to responsibly manage collaborative research that discovers, develops, and delivers health care solutions for service members, veterans, and the American public. The CDMRP fosters novel approaches to research and brings together diverse groups of stakeholders that otherwise may not collaborate. Co-location with the Army acquisition framework enables a low management rate, allowing >90% of appropriated dollars to be invested in research.

**Table 1:** CDMRP managed and supported programs and FY 2020 appropriations.

**CDMRP program**	**FY2020 appropriation, $ million**
Amyotrophic Lateral Sclerosis	$20.0
Alzheimer's	$15.0
Autism	$15.0
Bone Marrow Failure Disease	$3.0
Breast Cancer	$150.0
Breast Cancer Research Semipostal	TBD
Chronic Pain Management	$15.0
Combat Readiness Medical	$10.0
Duchenne Muscular Dystrophy	$10.0
Epilepsy	$12.0
Gulf War Illness	$22.0
Hearing Restoration	$10.0
Joint Warfighter Medical	$40.0
Kidney Cancer	$40.0
Lung Cancer	$14.0
Lupus	$10.0
Melanoma	$20.0
Military Burn	$10.0
Multiple Sclerosis	$16.0
Neurofibromatosis	$15.0
Orthotics and Prosthetics Outcomes	$15.0
Orthopedic	$30.0
Ovarian Cancer	$35.0
Pancreatic Cancer	$6.0
Neurotoxin Exposure Treatment Parkinson's	$16.0
Peer Reviewed Cancer (14 topics)	$110.0
Peer Reviewed Medical (44 topics)	$360.0
Prostate Cancer	$110.0
Rare Cancers	$7.5
Reconstructive Transplant	$12.0
Scleroderma	$5.0
Spinal Cord	$40.0
Tick-Borne Disease	$7.0
Tuberous Sclerosis Complex	$6.0
Vision	$20.0
**Additional supported DOD programs/projects**^**a**^	
Armed Forces Institute of Regenerative Medicine	TBD
Defense Medical Research and Development	$232.1
Psychological Health/Traumatic Brain Injury	$165.0
Small Business Innovation Research/Small Business Technology Transfer	TBD
Trauma Clinical	$10.0

a *Approximate funding of additional supported DOD programs/projects*.

The CDMRP's research management model includes targeted funding opportunity announcements and active engagement with patients, including veterans, living with health conditions. Veterans weigh in on program policy, investment strategy, and research focus. This model enables a two-way continuum between basic science and clinical application and promotes promising approaches to advance research and enhance healthcare in the veteran community. Using complementary funding opportunity announcements and synergistic funding mechanisms that encourage collaboration, the CDMRP and the VA have the capacity to better harness the power of research and evidence and make sure it is translated into action.

## CDMRP Program Cycle

**Figure 1:**
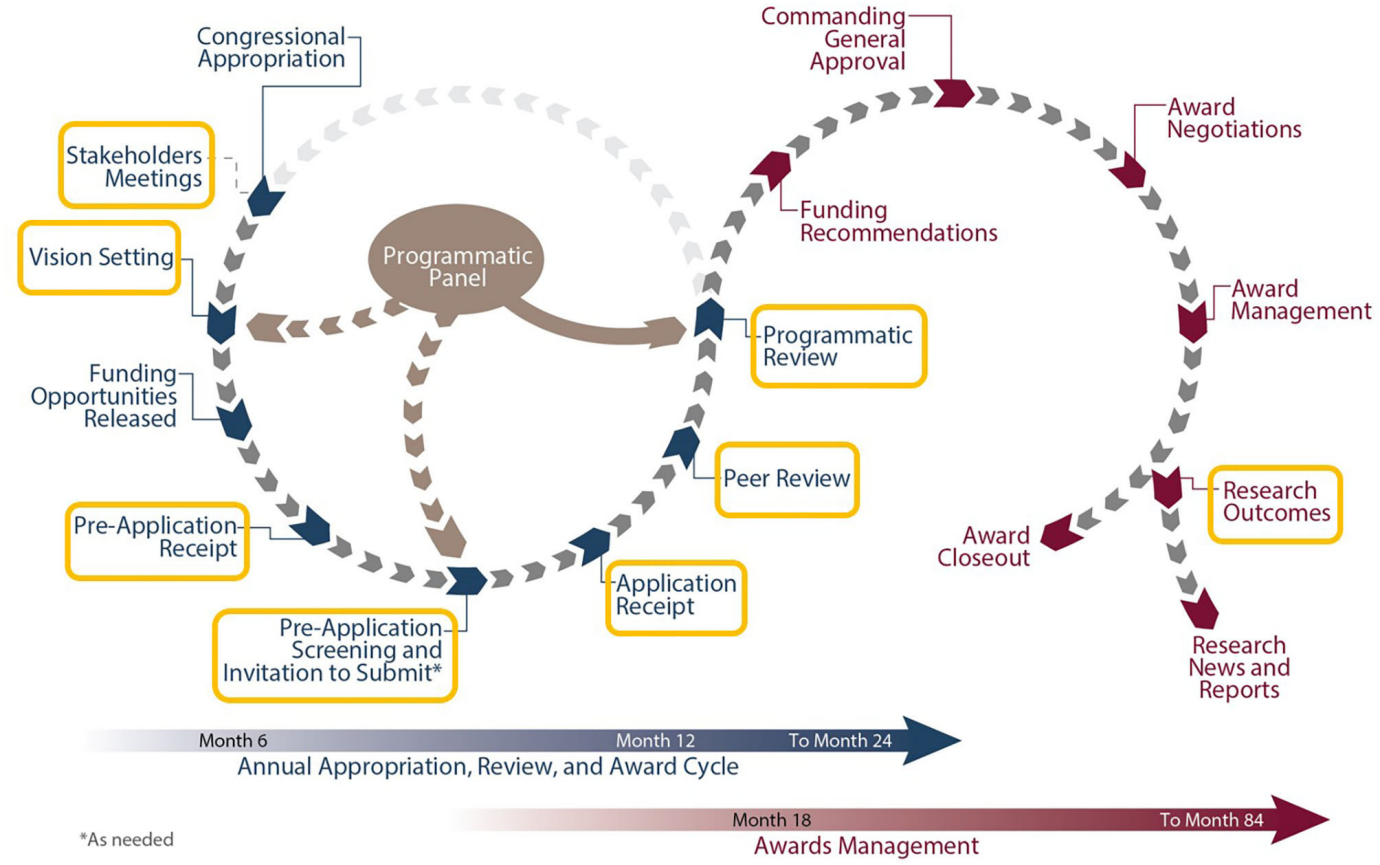
CDMRP program cycle highlighting opportunities for collaboration with the VA.

Each CDMRP-managed program has individually tailored goals and a distinct vision enabling funded research to meet the specialized needs of the research and patient communities. Several programs focus on mental health and other topics relevant to veteran health care, and opportunities for collaboration with the VA exist throughout the program cycle (see Figure 1).

The first major milestone in the cycle is the Vision Setting meeting, during which the Programmatic Panel convenes to assess the research landscape and develop an investment strategy. Each Programmatic Panel is comprised of a multidisciplinary group of research scientists, clinicians, foundations, industry, other federal agencies [such as the VA and National Institutes of Health (NIH)], and consumer advocates. For example, the Alcohol and Substance Abuse Disorders Research Program (ASADRP) Programmatic Panel consists of members from the U.S. Army, the National Institute on Alcohol Abuse and Alcoholism, the National Institute on Drug Abuse, and the Department of Veterans Affairs. Based on knowledge from their respective portfolios, the ASADRP Programmatic Panel recommended at a recent Vision Setting meeting an investment in multi-institutional research consortia involving teams of leading scientists and clinicians working together under one umbrella to identify pharmacotherapies that address alcohol and substance abuse disorders, with an emphasis on those related to TBI and PTSD in service members and veterans.

After establishing the program vision and investment strategy, Program Announcements are developed to solicit research applications that will make a significant and non-incremental impact (8). CDMRP uses a two-tier review process, recommended by the National Academy of Medicine, to identify research proposals that target program goals while also capturing the traditional strengths of scientific merit peer review systems. Initially, applications are evaluated for scientific and technical merit against criteria published in the funding opportunity. This first review is conducted by a scientific peer review panel consisting of scientists, clinicians, administrators from the VA and other stakeholder organizations, and consumers, independent of those serving on the Programmatic Panel. Scientific merit peer review is followed by a comparison-based programmatic review conducted by the Programmatic Panel. Although the ratings and evaluations of the peer reviewers are a key factor, programmatic reviewers do not automatically recommend funding applications that were highly rated in the scientific merit review process. Rather, they carefully compare applications ensuring proposals that have high potential to achieve the goals of the respective program and that contribute to a good portfolio balance are recommended for funding. This means that applications are not funded using an established “pay line.” At the final step of the program management cycle, funding recommendations are presented to the USAMRDC Commanding General for approval.

## CDMRP Hallmarks

A distinguishing CDMRP feature is the significant involvement from consumer advocates such as patients, survivors, family members, or caregivers impacted by the disease, condition, or injury throughout the program cycle. Applications are required to include an impact statement describing how the proposed research, if successful, will significantly advance the field and impact the patient advocate community (8). The potential impact of the proposed research is an important review criterion and consumer advocates hold equal weight to the clinicians, researchers, and other experts on the review panel. For example, consumer advocates involved with the ASADRP include active duty, veteran, or retired service members who have received treatment for alcohol and substance use disorders (ASUDs) related to PTSD and/or TBI and DOD or VA behavioral health providers. These consumer advocates play an important role in maintaining the focus of the ASADRP and provide a unique perspective on issues important to the end user community.

The CDMRP model facilitates investment in high-impact and high-risk/high-gain studies that other agencies may not fund. Unlike other federally funded agencies that receive funding in the President's budget each fiscal year, the CDMRP develops an investment strategy based on additional, individually-directed Congressional appropriations each fiscal year. Due to the nature of funding, multi-year awards are fully allocated up front, ensuring research projects are not put at funding risk. This also allows programs to shift focus year-to-year based on innovative advances in the field without the need to fulfill out-year budgets of previously selected awards. Additionally, the CDMRP uses novel, focused award mechanisms, directed at each disease, condition, or injury specified in Congressional language. For example, the Idea Award was developed by the Breast Cancer Research Program to support research with little or no preliminary data. Supporting early concepts and ideas complemented other Federal funding programs that required preliminary data, supported the next logical step, and were not disease- nor condition-focused (8). This CDMRP award mechanism moved research into new and unchartered territory in the 1990s and continues to be used by CDMRP programs today.

While industry, including pharmaceutical, biotechnology, and medical device firms, remains the largest funder of clinical trial research, the CDMRP's niche in this arena is the encouragement of preventive or therapeutic interventions that are in line with state of the art research and the priorities of the communities affected by the disease. For example, the ASADRP Pharmacotherapies for Alcohol and Substance Abuse (PASA) Consortium establishes pharmaceutical company partners who have compounds for ASUDs or PTSD available, but who need the complement of experts from the PASA to complete human studies to the U.S. Food and Drug Administration's (FDA) standards (9).

While all programs managed or supported by the CDMRP are focused on solutions for service members and the American public, three programs are particularly relevant to veteran health—the Gulf War Illness Research Program (GWIRP), the Psychological Health and Traumatic Brain Injury Research Program (PHTBIRP), and the ASADRP. From 2018 to 2019, the GWIRP, PHTBIRP and the ASADRP yielded ~500 publications, 20 patents/patent applications, and 50 research products. Furthermore, in a 2018 internal review of 10% of CDMRP-managed clinical trials funded between fiscal year 2008 and fiscal year 2015, 74% of closed trials met the CDMRP-defined criteria for success. These outcomes, as well as the unique aspects of the review and management model described herein, illustrate how CDMRP is able to translate important research results into medical solutions for patient communities.

## Collaborative Partnerships

The CDMRP has formed strong partnerships among the DOD, VA, and patient, scientific, and clinical communities to advance paradigm-shifting research and provide solutions that will lead to cures or improvements in care. CDMRP funding opportunities are open to VA researchers and clinicians as well as those from the military, academia, and the private sector. Inclusion of a military or VA investigator as an equal partner in the research is strongly encouraged in many CDMRP funding opportunities and the degree of this partnership is evaluated during the review process. Partnerships with the military and the VA are important to access relevant active duty and VA patient populations. In addition, the DOD and VA, with participation from the NIH, conduct a joint review and analysis of their portfolios annually.

The collaboration between the GWIRP and the VA is an example of a funding partnership that makes the best possible use of available resources in support of high-quality, veteran-focused research. The GWIRP was established in 2006 when Conferees directed the Secretary of the Army to utilize authorized funding to undertake research on Gulf War Illness (GWI) and to coordinate with similar activities at the VA. GWI is characterized by multiple, diverse symptoms not explained by established medical diagnoses or standard laboratory tests. The GWIRP is the only CDMRP program completely dedicated to veteran health, and frequent communication and collaboration occur between the VA and GWIRP Program Managers (PMs). Specific examples of such collaborations include GWIRP PM participation on VA-convened working groups, VA funding of GWIRP spin-off proposals, sharing of funding data through electronic coordination, and a recent GWI State of the Science conference co-hosted by the VA Office of Research and Development and the GWIRP. In addition, the GWIRP awarded a Clinical Trials Consortium in fiscal year 2017 that includes two of the VA War Related Illness and Injury Study Centers as recruitment sites, further demonstrating collaborative and optimal use of resources.

The CDMRP provides program and award management support for many other consortia that involve partnerships between the DOD, VA, and academia. These large awards bring together multidisciplinary, multi-organizational teams to tackle complex problems affecting service members and veterans such as PTSD, TBI, suicide, and ASUDs. An advantage of the consortium model is the ability to require collection of common data elements and aggregate data from numerous studies into a single data repository. The CDMRP strongly encourages the sharing of data and research resources generated from CDMRP-funded research, while protecting participant privacy, confidential and proprietary data, and intellectual property. The STRONG STAR Consortium to Alleviate PTSD (CAP), a jointly funded DOD-VA effort, and the Military Suicide Research Consortium (MSRC) are two CDMRP-managed consortia that house data which can be accessed by other researchers who are interested in conducting secondary analyses of de-identified data. Qualified investigators can access the STRONG STAR-CAP^1^ and MSRC^2^ websites for additional information on how to request access to data.

Strategic direction and oversight of consortia and other large awards is typically provided by Government Steering Committees or External Advisory Boards that include representatives from the DOD, VA, NIH, Centers for Disease Control and Prevention, and/or other relevant stakeholder agencies. This ensures that products resulting from these awards meet the end users' needs. Findings from several studies funded under the MSRC are referenced in the *VA/DOD Clinical Practice Guideline for The Assessment and Management of Patients at Risk for Suicide*, which directly impacts veteran mental health treatment (10).

These collaborative partnerships provide opportunity for greater return on investment and more expedited scientific advances. Coordination with the VA throughout the entire CDMRP program cycle helps ensure that both organizations avoid duplication of effort, stay abreast of developments in the field, and continue to foster collaborative opportunities.

## Opportunities for Synergy Across the Research Continuum

Research spans a continuum from discovery to clinical trials before the fielding and clinical application of knowledge and products. The CDMRP uses directed funding mechanisms, targeting specific steps along the continuum of research development, including basic, translational, and clinical research. These innovative funding opportunity announcements also complement research funding at the VA.

VA research funding mechanisms are similar to those used at the NIH and rely primarily on the individual research interests of VA intramural investigators (11). The CDMRP complements VA research through use of synergistic award mechanisms that are tailored to areas of greatest need. While one agency may be better positioned to fund only one component of a project (e.g., basic science), another agency may be better positioned to fund other components (e.g., clinical trial). From 2018 to 2019, for example, GWIRP, ASADRP, and PHTBIRP-funded investigators obtained follow-on funding from the DOD, NIH, VA, and other agencies on more than two dozen occasions.

This type of synergistic funding between the CDMRP and VA is evidenced through GWI treatment investigations. Preliminary analysis from a pilot trial supported by the GWIRP demonstrated, in a subset of veterans with GWI, that 100 mg of coenzyme Q10 (known as CoQ10 or Ubiquinone) improved general self-reported health and physical functioning (12). The results from this pilot trial supported the possibility of efficacy and were the basis for a larger, VA-sponsored Phase III trial initiated in 2017 of Ubiquinol, the reduced form of CoQ10. Parallel to the Phase III trial, the GWIRP funded biomarker analysis both before and after CoQ10 therapy using blood collections from subjects in the clinical trial. Potential biomarkers will be correlated with symptom clusters, illness severity, and predicting responders to the intervention. More recently, the GWIRP invested in a true replication trial, using the same high quality/bioavailability product and validated outcomes that previously showed benefit in the GWIRP-funded pilot study. The VA will use the collective results of these studies to inform Clinical Practice Guidelines and VA Formulary considerations for ill Gulf War veterans.

Within the PHTBIRP, research progress has been accelerated through studies seeking to translate existing interventions initially developed and evaluated with funding from the NIH, VA, or other agencies for military and veteran contexts. Examples include suicide, substance misuse, and sexual assault/harassment prevention interventions originally designed for use with college students and suicide treatment interventions designed for civilian medical settings adapted for use with military and veteran populations (13–15). Tailoring existing evidence-based interventions to meet a specific need significantly reduces the time required before an intervention is able to touch service members' and veterans' lives.

The ASADRP-funded PASA Consortium uses planning grants for clinical trials and full study research grants for pre-clinical trials. This is a novel risk mitigation strategy that allows them to leverage a relatively small appropriation to conduct complex/high risk research involving drug trials on human subjects. The purpose of a planning grant is to create a clinical development plan that includes study protocol development, regulatory pathway development, identification of study sites, pharmaceutical company support, determination of the need for additional studies by the FDA, and a study budget. Once this has been accomplished, the documents are submitted to the PASA and undergo a two-tier review before a funding recommendation is considered. Nine compounds are in pre-clinical trials for alcohol use disorder and PTSD while five other compounds are undergoing clinical review.

These and other CDMRP funding opportunities demonstrate the value of leveraging and maximizing federal dollars to support veteran health. Such synergies can significantly accelerate progress, thus shrinking the pipeline for development of new therapeutics, new diagnostics, and changes in the standard of care.

## Conclusion

Multi-organizational, multidisciplinary research partnerships are needed to address the health burdens faced by veterans. Emerging results have the potential to be most impactful if they continue to feed into the two-way continuum between basic science and clinical application to inform patient care and future research priorities.

The CDMRP management model has many integrated activities that rely on partnerships with the VA to ensure translation of basic research to clinical applications. Additionally, the CDMRP fully integrates consumer advocates, including veterans with significant health issues, in program policy, investment strategy, and research focus. Many CDMRP funding mechanisms promote the translation of science from discovery through different development stages, including the transition to clinical studies and product development. Collaborating with the VA early in the development of the program strategy and funding mechanism facilitates translation of innovative and impactful technologies funded by the VA and DOD. This creates new medical solutions for service members, veterans, and the American public. Emphasizing collaboration and stepwise translation may help funding agencies facilitate a seamless transition, capitalize on efficiencies, and reduce costs. Such efforts are integral to improving the mental health of the veteran population and advancing the field.

To learn more about the CDMRP and its programs or to receive funding opportunity notifications by e-mail, please visit https://cdmrp.army.mil.

## Author Contributions

ML and KL participated in conceptualization of the article, drafting, and final editing. RS and SD participated in drafting and reviewing the article. All authors approved the final version of the manuscript to be published and agreed to be accountable for all aspects of the work.

## Conflict of Interest

The authors report no actual or potential conflicts of interest with regard to this article; however, the authors note that they are Department of Army employees affiliated with the CDMRP.

## Footnotes

^1^https://patriot.uthscsa.edu/strongstar/subs/rpinfo.asp?prj=12

^2^https://msrc.fsu.edu/members/request-access-msrc-database
